# Intron Dynamics in Ribosomal Protein Genes

**DOI:** 10.1371/journal.pone.0000141

**Published:** 2007-01-03

**Authors:** Maki Yoshihama, Hung D. Nguyen, Naoya Kenmochi

**Affiliations:** Frontier Science Research Center, University of Miyazaki, Kiyotake, Miyazaki, Japan; South African National Bioinformatics Institute, South Africa

## Abstract

The role of spliceosomal introns in eukaryotic genomes remains obscure. A large scale analysis of intron presence/absence patterns in many gene families and species is a necessary step to clarify the role of these introns. In this analysis, we used a maximum likelihood method to reconstruct the evolution of 2,961 introns in a dataset of 76 ribosomal protein genes from 22 eukaryotes and validated the results by a maximum parsimony method. Our results show that the trends of intron gain and loss differed across species in a given kingdom but appeared to be consistent within subphyla. Most subphyla in the dataset diverged around 1 billion years ago, when the “Big Bang” radiation occurred. We speculate that spliceosomal introns may play a role in the explosion of many eukaryotes at the Big Bang radiation.

## Introduction

Many spliceosomal introns, which are non-coding DNA sequences, exist in eukaryotic nuclear genes. Their role in the genome, however, remains poorly understood. From the view of eukaryotic evolution, it is very important to know why exon/intron structures of genes differ across species and what the effects of intron gain and loss are. In order to clarify these issues, we must first reconstruct the process of intron gain and loss during eukaryotic evolution. This task became possible recently with the availability of many completely sequenced genomes. In a representative study, Rogozin *et al.* [1] compiled a dataset of 684 gene orthologs from eight eukaryotes and used a maximum parsimony method to infer the evolution of introns in this dataset. The results of applying maximum likelihood methods to the same dataset were reported later [2]–[4]. Although the number of species in the dataset is not very large and the different methods inferred different patterns of intron gain and loss, it became clear that: (i) from 15% to 25% of present-day introns were already present in the last common ancestor of plantae, metazoa, and fungi, and (ii) many introns were gained after this divergence [1]–[4].

We have recently compiled a dataset of ribosomal protein (RP) genes [5]. RP genes offer several advantages for studying intron evolution [6]–[8]. First, they exist in all species and, as they are involved in the vital process of translation, they are well conserved throughout evolution [9], [10]. Thus, it is fairly easy to compare intron positions in RP genes across a wide range of distantly diverged species. Second, there are a large number of conserved RP gene families. For instance, 79 distinct RPs are found in humans and of these 79, 78 are also found in yeast. Third, introns also exist in RP genes of very deep-branching eukaryotes that harbor very few introns, such as *Giardia lamblia* [11], [12]. With these advantages, we expect that RP genes will become a powerful tool for discovering the roles of spliceosomal introns.

## Results

### Compilation of the dataset and phylogenetic analysis

**Figure 1:**
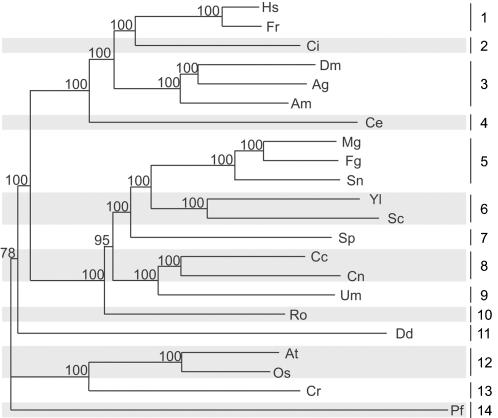
The Phylogenetic Tree of the 22 Eukaryotes in the Dataset. All conserved amino acids regions in 76 RP genes were used to generate the tree. The tree with bootstrap values in percentages was built using the Seqboot (1000 replicates), Protdist, Neighbor, and Consense programs of the PHYLIP package [28]. The taxonomy is based on that of NCBI (http://www.ncbi.nlm.nih.gov/Taxonomy/) and ITIS (http://www.itis.usda.gov/index.html). Hs, *Homo sapiens*; Fr, *Fugu rubripes*; Ci, *Ciona intestinalis*; Dm, *Drosophila melanogaster*; Ag, *Anopheles gambiae*; Am, *Apis mellifera*; Ce, *Caenorhabditi elegans*; Mg, *Magnaporthe grisea*; Fg, *Fusarium graminearum*; Sn, *Stagonospora nodorum*; Yl, *Yarrowia lipolytica*; Sc, *Saccharomyces cerevisiae*; Sp, *Schizosaccharomyces pombe*; Cc, *Coprinus cinerea*; Cn, *Cryptococcus neoformans*; Um, *Ustilago maydis*; Ro, *Rhizopus oryzae*; Dd, *Dictyostelium discoideum*; At, *Arabidopsis thaliana*; Os, *Oryza sativa*; Cr, *Chlamydomonas reinhardtii*; Pf, *Plasmodium falciparum;* 1, vertebrata; 2, urochordata; 3, insecta; 4, nematoda; 5, pezizomycotina; 6, saccharomycotina; 7, schizosaccharomycetes; 8, hymenomycetes; 9, ustilaginomycetes; 10, zycomycetes; 11, mycetozoa; 12, magnoliophyta; 13, chlorophyta; 14, apicomplexa.

We compiled a dataset of 76 RP gene orthologs from 22 eukaryotes. The phylogenetic tree of these 22 species is depicted in Figure 1. These 22 species belong to four kingdoms, metazoa, fungi, protozoa, and plantae, and cover 14 different subphyla. The conserved regions of this dataset included 2,961 introns located at 1,182 different positions. To the best of our knowledge, this is the first time a dataset with this many gene families and species has been used for studying intron evolution.

### Patterns of intron gain and loss in 22 species

**Figure 2:**
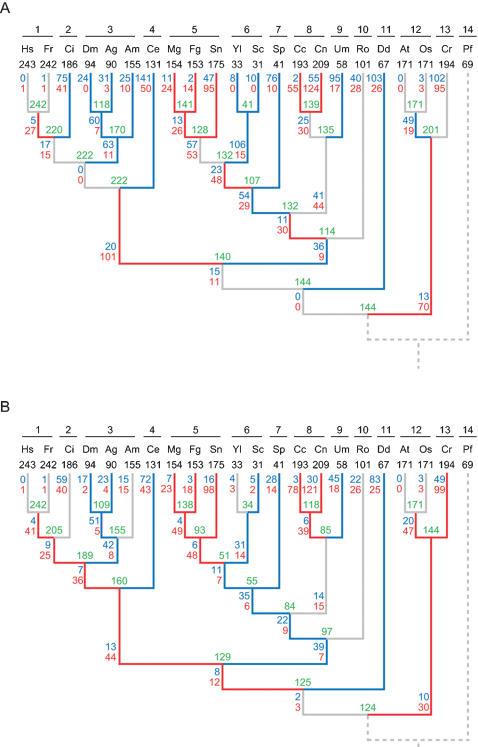
Patterns of Intron Gain and Loss. (A) Likelihood results. (B) Parsimony results. Numbers of introns present in modern species (known) are in black. Numbers of introns present in ancestors (estimated) are in green. Numbers of gains and losses (estimated) are in red and blue, respectively. Branches that experienced >1.5 gains per loss are shown in red and those that experienced >1.5 losses per gain are in blue. Abbreviations are the same as in Figure 1.

We first used our recently developed maximum likelihood (ML) method [4] to infer the process of intron gain and loss (Figure 2A). We also used a maximum parsimony method to validate the result of the ML method, because the ML method may produce unreliable results when the data sample is small (Figure 2B). Since the two results show similar patterns of intron gain and loss in most subphyla of the dataset (the largest differences are in the two plant subphyla), the results from the ML method were used for subsequent analyses.

The most significant feature in Figure 2 is that species belonging to a given subphylum show similar trends of intron gain and loss. For example, all three species in insecta (subphylum 3 in Figure 2) show trends toward decreasing introns, whereas all three species in pezizomycotina (subphylum 5) show trends toward increasing introns. There is, however, no consensus trend of intron gain and loss among species of a given kingdom. This fact is most notable in the fungus kingdom. The subphyla pezizomycotina (subphylum 5) and hymenomycetes (subphylum 8) trended toward increasing introns, whereas the three other subphyla [saccharomycotina (subphylum 6), schizosaccharomycetes (subphylum 7), and ustilaginomycetes (subphylum 9)] showed the opposite trend. There is also no consensus trend of intron gain and loss among species of a given cell complexity (i.e., unicellular or multicellular). For multicellular species, introns decreased in insecta (subphylum 3) and nematoda (subphylum 4) but increased in *Coprinus cinerea* and pezizomycotina (mycelium, subphylum 5). Likewise, introns increased in the unicellular species *Cryptococcus neoformans* but decreased in saccharomycotina (subphylum 6) and schizosaccharomycetes (subphylum 7). Similar results were also obtained in an analysis using 13 additional fungal species (data not shown).

### Intron gain and loss and the speciation of eukaryotes

**Figure 3:**
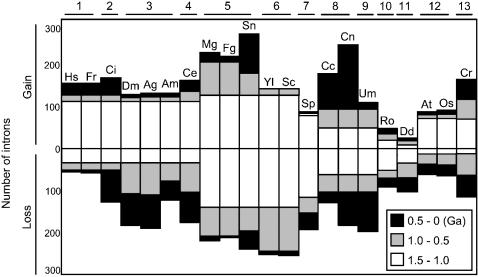
Intron Gains and Losses Over Time. The numbers of intron gains and losses are divided into three periods of time: 0.5–0 Ga (black); 1.0–0.5 Ga (gray), and 1.5–1.0 Ga (white). The timescale is based on that of Hedges and colleagues [13], [14]. Ga, billion years ago; other abbreviations are the same as in Figure 1.

Figure 3 shows the numbers of intron gains and losses divided into three periods of time (the timescale is based on that of Hedges and colleagues [13], [14]). The numbers of intron gains and losses are similar for each species in a given subphylum containing multiple species, but they clearly differ across subphyla. For example, during the period from 1.5 to 0 billion years ago (Ga), vertebrata (subphylum 1) had more intron gains than losses (∼155 gains versus ∼58 losses on average). In contrast, insecta (subphylum 3) had more intron losses than gains (∼165 losses versus ∼132 gains on average). In particular, the excess of intron losses over gains in *Drosophila melanogaster* and* Anopheles gambiae* were very strong during the period from 1.0 to 0 Ga (∼151 losses versus ∼20 gains on average).

During the period from 1.5 to 0 Ga, the species with the most intron gains was *Stagonospora nodorum* (275 introns), and the species with the fewest gains was *Dictyostelium discoideum* (26 introns). During this period, the species with the most intron losses was *Saccharomyces cerevisiae* (255 introns), and the species with the fewest losses was *Homo sapiens* (57 introns). The patterns of intron gain and loss were most complicated in fungi. The number of intron losses in *Magnaporthe grisea* since the crown ancestor was 220, whereas the number of gains was 230. During the period from 1.5 to 0 Ga, fungal species in average experienced 2.2 intron gains and 2.6 intron losses per gene (as 168 gains and 198 losses in 76 genes). In contrast, humans experienced 2.0 intron gains and 0.8 intron losses per gene (as 155 gains and 57 losses in 76 genes).

## Discussion

In this study, we have analyzed intron dynamics in 76 RP gene families from 22 species. So far, there has been no analysis that used such a large dataset with many gene families and species. On one hand, the results inferred from the RP genes are mostly in-line with previous studies using fewer species. For example, when the same maximum likelihood was used, the trends of intron gains and losses in seven species of the 684-ortholog dataset [1], [4] are similar to those presented here. In addition, the finding that species with short generation time have a trend of losing their introns [15], [16] can also be observed in this study. On the other hand, RP genes are easier to extend to more species because they are not too large in number and are highly conserved. Thus, we believe that RP genes are a suitable dataset for studying the evolution of introns.

When the intron dynamics in RP genes from 22 species were viewed together, it becomes clear that the trends of intron gains and losses differ across species in a given kingdom but are similar within subphyla. It has been proposed that most subphyla in our dataset diverged around 1 Ga [13], when an explosion of eukaryotes occurred (the Big Bang hypothesis) [17]. Furthermore, Babenko et al. [18] suggested that intensive intron gains and losses occurred during limited time spans, perhaps coinciding with major evolutionary transitions. Therefore, we speculate that there may be a relationship between the process of intron gain and loss and the divergence of species at the Big Bang radiation.

Several roles have been proposed for spliceosomal introns. One of these is to increase the variation of proteins through alternative splicing [19]–[21]. Because alternative splicing is found in both multicellular plants and animals, it was proposed to be a necessary tool for the evolution of eukaryotic complexity [22]. However, although 40% to 50% of human genes are currently estimated to have splicing variations [19]–[21], genes without splicing variations also exist. In fact, almost no alternative splicing of human RP genes has been reported so far.

If alternative splicing is not the only role of introns, what are other roles? We speculate that there is a relationship between introns and non-coding RNAs (ncRNAs) and, through the process of intron gain and loss, introns may affect the variation of expression of ncRNAs to become a force for the speciation of eukaryotes. There are several lines of supporting evidence for this speculation. First, some ncRNAs are transcribed from introns in protein coding genes [23]. In fact, many small nucleolar RNAs (snoRNAs) are encoded by introns of RP genes in vertebrates [5], [6]. These snoRNAs are expressed through the splicing of the pre-mRNAs of RP genes and act as RNA molecules after they are spliced out from the introns [24], [25]. Second, the finding that adaptive changes to non-coding DNA might have been more common in the evolution of *D. melanogaster* [26] suggests that introns may be functional not only in vertebrates but also in other eukaryotes. Third, ncRNAs have been proposed to be involved in the development of multicellular species [22], [23]. Therefore, we speculate that eukaryotic introns may function as carriers for ncRNAs and, through the process of intron gain and loss, may affect the level and variation of expression of ncRNAs.

## Materials and Methods

### Compilation of the dataset

The RP genes of 8 eukaryotes (*H. sapiens, Ciona intestinalis, D. melanogaster, Caenorhabditi elegans, Schizosaccharomyces pombe, S. cerevisiae, Arabidopsis thaliana, and Plasmodium falciparum*) were taken from the manually curated Ribosomal Protein Gene database (RPG, http://ribosome.med.miyazaki-u.ac.jp) [5]. The RP gene sequences of other species were collected by performing a BLAST search using human RP genes as queries. Their gene structures were then manually constructed both by using annotation (if available) and by aligning their sequences with those of other species. Finally, the constructed genes were used as queries to a BLAST search against the human genes to ensure that they and the human RP genes are reciprocal best hits. Genome homepages for the different species we investigated are as follows: *H. sapiens, A. gambiae*, and *Apis mellifera* (ENSEMBL, http://www.ensembl.org/index.html); *D. melanogaster* (FlyBase, http://flybase.net/); *C. elegans* (WormBase, http://www.wormbase.org/); *S. pombe* (http://www.sanger.ac.uk/Projects/S_pombe/); *S. cerevisiae* (SGD, http://www.yeastgenome.org/); *M. grisea, Fusarium graminearum, S. nodorum, C. cinerea, C. neoformans, Ustilago maydis*, and *Rhizopus oryzae* (Fungal Genome Initiative, http://www.broad.mit.edu/annotation/fgi/); *Yarrowia lipolytica* (NCBI, ftp://ftp.ncbi.nih.gov/genomes/Fungi/Yarrowia_lipolytica_CLIB99); *D. discoideum* (dictyBase, http://www.dictybase.org/); *A. thaliana* (TAIR, http://www.arabidopsis.org/info/agi.jsp); *Oryza sativa* (TIGR, http://www.tigr.org/tdb/e2k1/osa1/); *C. intestinalis, Chlamydomonas reinhardtii*, and *Fugu rubripes*, (JGI, http://genome.jgi-psf.org/euk_cur1.html); and *P. falciparum* (http://www.sanger.ac.uk/Projects/P_falciparum/). When a gene existed in multiple copies in a given species, the copy with the most introns was used. The sequences of the 76 orthologs of RP genes are available at http://ribosome.miyazaki-med.ac.jp/.

### Construction of intron presence/absence matrix and phylogenetic tree

Multiple sequence alignments for each of the gene orthologs were built using ClustalW [27], and an *ad hoc* program was written in the C programming language to extract the intron presence/absence matrix (Dataset S1) and conserved amino acid regions of these alignments. The conserved amino acid regions were then concatenated together and the Seqboot (1000 replicates), Protdist, Neighbor, and Consense programs of the PHYLIP package [28] were used to build a neighbor-joining tree with bootstrap values (Figure 1).

### Inference of intron evolution

Two methods, maximum likelihood [4] and maximum parsimony [7], were used for inferring the patterns of intron gains and losses. Both methods take as input the intron presence/absence matrix and the phylogenetic tree (with *P. falciparum* as the out-group). In a previous analysis of intron evolution [4], we have shown that there are ∼42,000 possible sites for intron insertion, but the number of introns at the root of the phylogenetic tree is rather small (we estimated it to be ∼450, which equals the number of introns in *P. falciparum*). Therefore, the cost function in the maximum parsimony method was modified as follows:
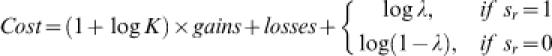
where *gains* and *losses* are the numbers of intron gains and losses; *K* is the ratio between rate of loss and rate of gain; *λ* shows the fraction of introns present at the root of the phylogenetic tree; and *s_r_* = 0 or 1 means intron absence or presence at the root. The values *K* = 500 and *λ* = 0.01 were used here.

## Supporting Information

Dataset S1 Matrix of the Entire Set of Intron Presences and Absences.
(0.03 MB TXT)
